# An integrated three-stream network model for discriminating fish feeding intensity using multi-feature analysis and deep learning

**DOI:** 10.1371/journal.pone.0310356

**Published:** 2024-10-21

**Authors:** Yanbin Dong, Shilong Zhao, Yuqing Wang, Kewei Cai, Hongshuai Pang, Ying Liu

**Affiliations:** 1 College of Marine Technology and Environment, Dalian Ocean University, Dalian, Liaoning Province, China; 2 Key Laboratory of Environment Controlled Aquaculture (Dalian Ocean University) Ministry of Education, Dalian, Liaoning Province, China; 3 College of Mechanical and Electronic Engineering, Dalian Minzu University, Dalian, Liaoning Province, China; 4 College of Information Engineering, Dalian Ocean University, Dalian, China; 5 College of Biosystems Engineering and Food Science, Zhejiang University, Hangzhou, Zhejiang Province, China; Dalian Maritime University, CHINA

## Abstract

Feed costs constitute a significant part of the expenses in the aquaculture industry. However, feeding practices in fish farming often rely on the breeder’s experience, leading to feed wastage and environmental pollution. To achieve precision in feeding, it is crucial to adjust the feed according to the fish’s feeding state. Existing computer vision-based methods for assessing feeding intensity are limited by their dependence on a single spatial feature and manual threshold setting for determining feeding status constraints. These models lack practicality due to their specificity to certain scenarios and objectives. To address these limitations, we propose an integrated approach that combines computer vision technology with a Convolutional Neural Net-work (CNN) to assess the feeding intensity of farmed fish. Our method incorporates temporal, spatial, and data statistical features to provide a comprehensive evaluation of feeding intensity. Using computer vision techniques, we preprocessed feeding images of pearl gentian grouper, extracting temporal features through optical flow, spatial features via binarization, and statistical features using the gray-level co-occurrence matrix. These features are input into their respective specific feature discrimination networks, and the classification results of the three networks are fused to construct a three-stream network for feeding intensity discrimination. The results of our proposed three-stream network achieved an impressive accuracy of 99.3% in distinguishing feeding intensity. The model accurately categorizes feeding states into none, weak, and strong, providing a scientific basis for intelligent fish feeding in aquaculture. This advancement holds promise for promoting sustainable industry development by minimizing feed wastage and optimizing environmental impact.

## Introduction

Feed assumes a pivotal role in aquaculture, with meticulous regulation of feeding quantities emerging as a crucial determinant in curtailing breeding expenses and augmenting breeding efficiency. The feeding process during fish cultivation primarily hinges on the acumen of breeding technicians. They employ either manual techniques or automated feeding apparatus to ensure consistent and quantitative feeding. Nevertheless, this strategy may precipitate issues such as underfeeding or overfeeding. Insufficient feeding can negatively influence the growth rate of fish, while surplus feeding culminates in food wastage and escalated breeding costs. Furthermore, residual bait in the aquatic milieu engenders ammonia nitrogen and harmful nitrate compounds. This occurrence could disrupt the optimal conditions requisite for the flourishing growth of fish. Alterations in fish physiology, feeding necessities, and organism behavior serve as direct indicators of the influence of environmental factors [1]. Feeding behavior, defined as the behavioral response of fish during the feeding process, forms a critical aspect of aquaculture, reflecting the dietary needs of the aquatic species. The identification of the feeding state entails classifying the feeding motion in the fish’s current state [2]. The recognition of a fish’s robust feeding state denotes existing nutritional needs, while a weaker state implies reduced feeding requirements. As a result, the accurate determination of the feeding status enables the evaluation of their dietary needs, promotes timely modifications in feeding quantities, and curtails feed wastage. This rigorous approach has become an essential component of feeding optimization in modern aquaculture practices [3].

At present, certain individuals utilize conventional computer vision technology to tackle the challenge of selecting feeding nodes for farmed fish. Being non-contact and non-invasive, computer vision technology has emerged as a cornerstone for propelling intelligent aquaculture due to its high efficiency, precision, and user-friendly interface [4]. The application of computer vision technology to discern the feeding status of fish populations and integrate fish biomass for forecasting and adjusting feeding quantities is crucial for diminishing aquaculture costs and enhancing fish welfare. Researchers worldwide have harnessed computer vision technology for the exploration of fish behavior [5–9], accomplishing the recognition of a variety of fish behaviors, encompassing hunger [10] and abnormalities [11]. In studies centered on feeding behavior recognition [12, 13], the strategy adopted by Zhou et al. (2017) [13] entailed the use of support vector machine (SVM) and gray-level co-occurrence matrix (GLCM) methods. By extracting fish centroids and constructing a Delaunay triangulation network, the computation of the clustering index for fish feeding behavior is realized. This method enables the identification and quantification of feeding behavior with heightened precision. Guo et al. (2018) [14] extracted shape and textural attributes from fish visual data and utilized the backpropagation neural network algorithm to discern and identify the feeding status of aquatic organisms. Yu et al. (2021) [15] employed Harris corner detection in conjunction with the Lucas-Kanade optical flow method to procure feature points and velocity information from images capturing fish feeding behavior. They devised a model that amalgamates standard behavior feature matrices with unique behavior detection to accomplish the recognition of fish feeding and other specific behaviors.

Presently, computer vision methods can only scrutinize a single spatial feature and rely on manually set thresholds to ascertain the feeding state, leading to limitations, impracticality, and other issues for specific scenarios and objectives. To surmount these challenges, the introduction of Convolutional Neural Network (CNN) based on deep learning theory can effectively mitigate these limitations. CNN can directly utilize the image as input to identify microscopic features, obviating the need for manual feature extraction and facilitating swift and precise discrimination of the feeding status of farmed fish. Deep learning autonomously extracts low-level features and engenders more abstract high-level representations, eliminating the necessity for a distinct target segmentation process and achieving enhanced accuracy and adaptability. Inherently, Deep learning models capture highly nonlinear and complex features through multi-layer sequences for self-learning [16, 17], steering the network towards an optimal state for precise classification. Convolutional Neural Networks (CNNs) demonstrate automatic learning of target features and boast a significant modeling capacity, exhibiting exceptional performance across a variety of domains such as agriculture, medicine, among others [18, 19]. Indeed, these methodologies have demonstrated efficacy in aquaculture, particularly in the identification of fish species and quality grading [20, 21]. In the context of identifying fish feeding behavior, Måløy et al. (2019) [21] pioneered a two-stream recursive network that amalgamates a spatial network, a 3D convolutional motion network, and an LSTM recursive classification network. This avant-garde approach attains a remarkable accuracy of 80% in predicting salmon feeding behavior. Zhou et al. (2019) [6] devised a model employing the convolutional neural network under controlled laboratory conditions to discern fish-feeding states, achieving a noteworthy accuracy of 90%. Zhang et al. (2023) [22] introduced an enhanced MobileNetV3 network, referred to as the Multi-Scale Information Fusion Network (MSIF-MobileNetV3), to analyze fish feeding behavior, attaining an accuracy of 96.4%. However, a challenge emerges when relying exclusively on CNN for image discrimination; CNN tends to concentrate on numerous detailed features, potentially leading to data redundancy, escalated computational complexity, and diminished accuracy. Therefore, further comprehensive analysis is crucial to determine the significance of deep learning networks in extracting image features and to explore how to select and provide the critical features required for network learning, thereby significantly enhancing the accurate recognition of fish feeding status.

To address the aforementioned challenges, we proposed a three-stream network model specifically designed to discern fish-feeding behavior, capitalizing on computer vision technology and Convolutional Neural Network (CNN). Given that the feeding process evolves over time, a comprehensive analysis of the image’s temporal and spatial characteristics is paramount for accurately capturing shifts in fish feeding intensity within the breeding environment and assessing feeding efficacy. In the preliminary stage, we utilize optical flow, binarization, and Gray-Level Co-occurrence Matrix (GLCM) techniques to process the original image. This enables the extraction of temporal, spatial, and statistical features from the video data. Subsequently, a CNN grounded in deep learning is employed to discriminate the feeding state. The classification outcomes of the three networks are subsequently fused using a late fusion approach [23], specifically fusion at the prediction score level. This strategy involves training three models independently, each generating a prediction score. The results from the three models are consolidated to derive the final discrimination outcomes for the intensity of fish feeding. Grounded in deep learning networks, the proposed model enables multi-feature fusion computations and automatic extraction of target features. It exhibits robust generalization, facilitating the execution of classification tasks for different targets by training the network with diverse datasets. The model adopts an end-to-end approach, enhancing its practicality and addressing deficiencies in existing computer vision technology. Ultimately, our method achieves a remarkable accuracy of 99.3%, effectively distinguishing the feeding status of fish. This research provides technical support for intelligent and accurate feeding, cost reduction, efficiency enhancement, water pollution reduction, and ultimately, the healthy growth and sustainable development of fish.

## Materials and methods

### Experimental environment

The experiment was conducted at the Aquaculture Facilities and Equipment Engineering Research Center of Dalian Ocean University. The experimental fish utilized were pearl gentian grouper (*♀ Epinephelus fuscoguttatus × ♂ Epinephelus lanceolatus*), averaging a size of 100 g. Furthermore, the Dalian Ocean University Experimental Animal Ethics Committee exempted this study from ethical review and approval, given that the fish used in this research are solely for data collection, undergo routine feeding procedures, and do not cause any harm to the fish.

**Fig 1:**
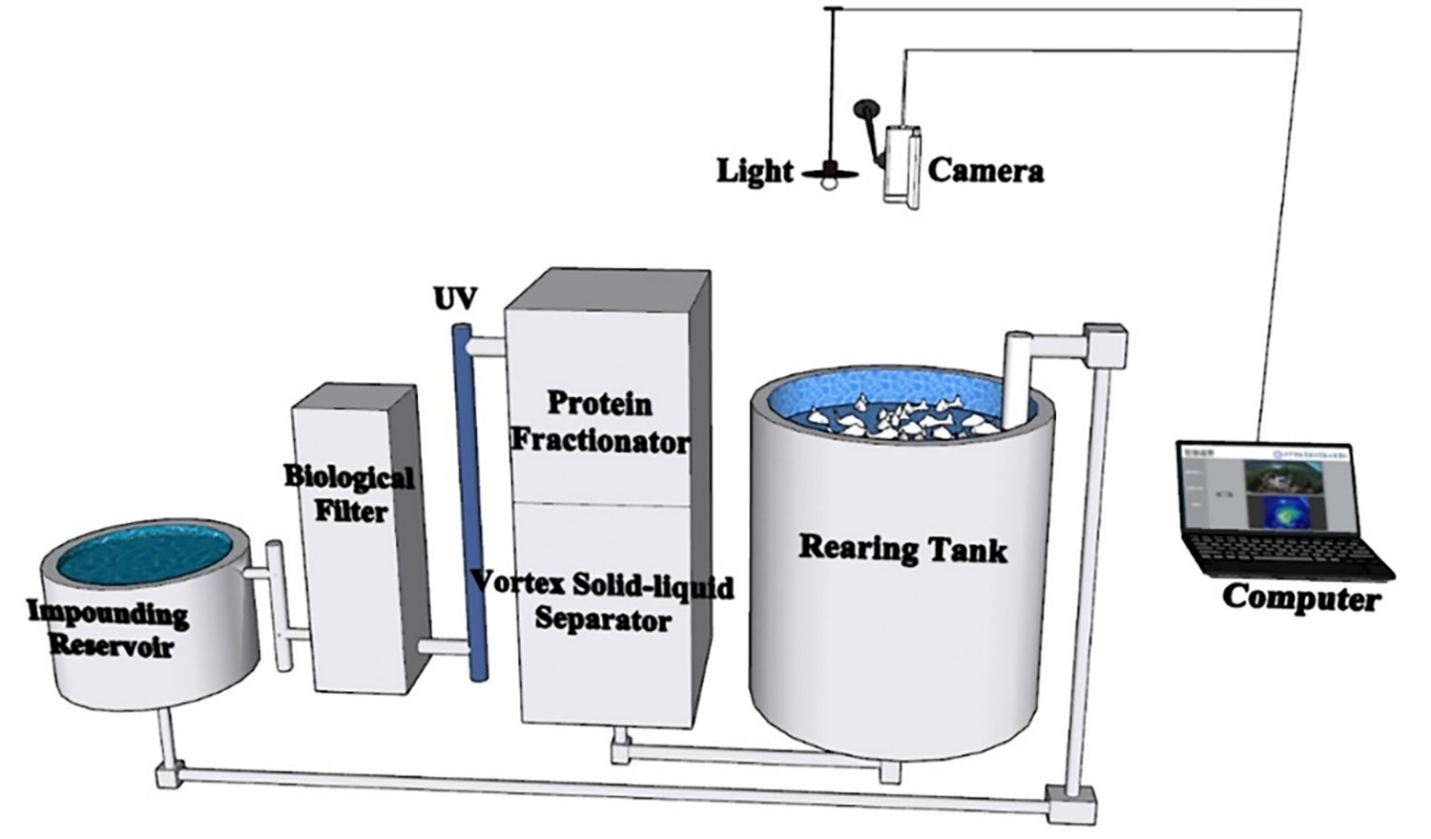
Experimental system diagram.

The aquaculture system, depicted in Fig 1, employed a comprehensive experimental system of circulating water. Each culture system comprised a culture barrel, a vortex solid-liquid separator, a protein separator, a crawler microfilter, a thermostat, a biological filter, an ultraviolet sterilization device, and an oxygen-increasing device. The culture barrel, constructed of blue plastic, has an upper part approximately cylindrical in shape and a lower part approximately conical. It has a diameter of 0.93 m, with the water depth meticulously maintained at 1.00 m, a sufficient culture water volume of 0.64 m^3^, and a water intake of 0.8 m^3^ per hour. The aquaculture system automatically discharges sewage and replenishes the source water, which is natural seawater precipitated. The light source is a full-spectrum LED lamp with an automatic control switch. The illumination time spans from 7:30 to 19:30, and the maximum light intensity in the breeding barrel is 372 lx. The camera system, controlled by a computer, captures images from a fixed position. The camera, positioned 0.5 m away from the water surface, can capture the entire water surface image and record the complete video from one minute before feeding to one minute after the bait is completely consumed. Feeding was conducted at intervals of 6 hours per day, with the feed delivered in several batches.

### Data preprocessing

**Fig 2:**
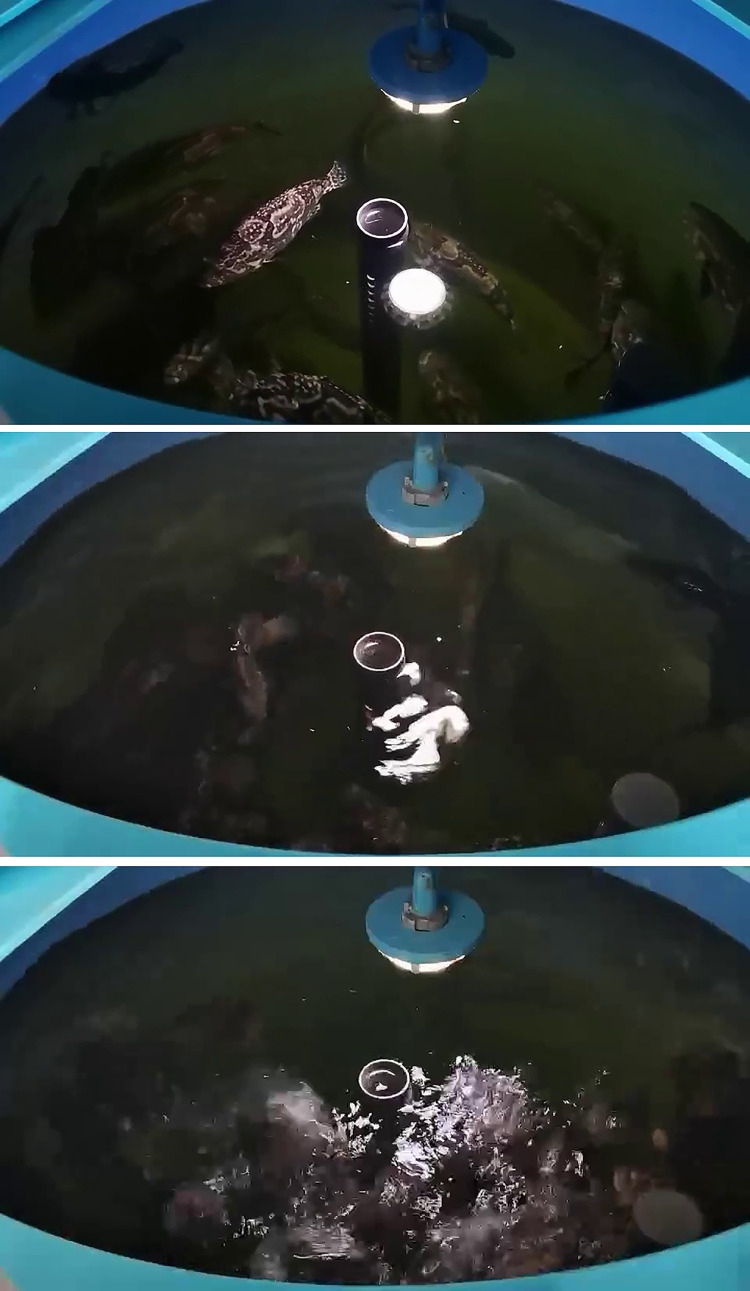
RGB images of three different feeding states. (A) None Feeding State. (B) Weak Feeding State. (C) Strong Feeding State.

Throughout the fish feeding period, we gathered 70 comprehensive feeding videos for editing, each boasting a resolution of 480 x 270 pixels. Observing the fish activity during feeding, we manually classified and edited the videos into distinct feeding status categories, each enduring approximately 7 seconds. These categories were partitioned into three classes: strong feeding status, weak feeding status, and no feeding status. The feeding status classification standard [24] is elaborated in Table 1, with each category encompassing 186, 217, and 216 short videos, respectively, culminating in a total of 619 feeding status videos. The videos of the three states were segmented and stored at a rate of 24 frames per second, yielding a total of 73,474 RGB images. This set comprises 22,888 non-feeding states, 23,728 strong feeding states, and 26,858 weak feeding states. The RGB images corresponding to each feeding state are illustrated in Fig 2. To compile a comprehensive dataset, we divided the original dataset into training, test, and validation sets, constituting 70%, 20%, and 10% of the total dataset, respectively. The proportion of the three feeding states in each dataset was consistently maintained.

**Table 1:** Classification criteria of feeding state intensity.

Feeding State Intensity	Standard Of Classification
None	The fish did not respond to food, and the water was calm.
Weak	The fish eat only the bait that falls in front of them or move to feed; after eating back to the original position, and the water surface has obvious fluctuation.
Strong	The fish moves freely between the food, eats all the available food, and the fierce swimming stirs up the water.

### A three-stream network for identifying the feeding status of cultured fish

**Fig 3:**
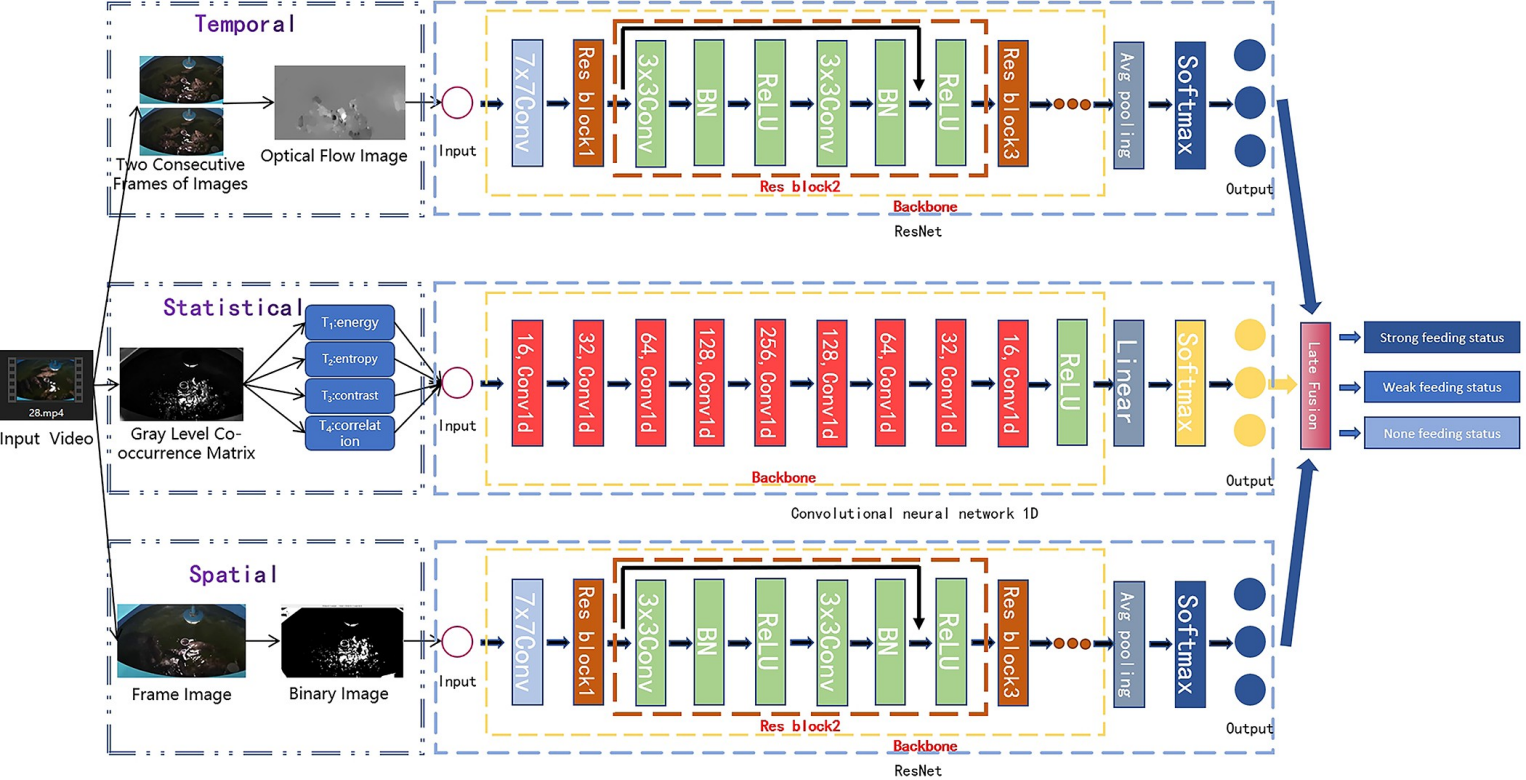
Model structure diagram.

Given that the feeding process unfolds over time, it is imperative to analyze the com-prehensive temporal and spatial characteristics of the image. This is vital for accurately capturing shifts in fish feeding intensity within the breeding environment and assessing feeding efficacy. In the preliminary stage, we employ optical flow, binarization, and Gray-Level Co-occurrence Matrix (GLCM) techniques to process the original image. This facilitates the extraction of temporal, spatial, and statistical features from the video data. Subsequently, a Convolutional Neural Network (CNN) grounded in deep learning is utilized to discern the feeding state. The classification outcomes of the three networks are then fused using a late fusion approach [23], specifically fusion at the prediction score level. This strategy involves training three models independently, each generating a prediction score. The results from the three models are amalgamated to derive the final discrimination outcomes for the intensity of fish feeding. The network structure is illustrated in Fig 3.

#### Classification network of temporal features based on optical flow method

Through empirical observation, we have ascertained that the process of fish feeding adheres to a chronological sequence. As a result, we utilize the optical flow method to process images, capturing the temporal fluctuations in fish behavior during feeding. Moreover, we have selected ResNet50 as our backbone network, using images processed by the optical flow method as network input for training and accuracy testing. This approach empowers us to distinguish and classify the feeding intensity of fish from the vantage point of temporal characteristics.

**Fig 4:**
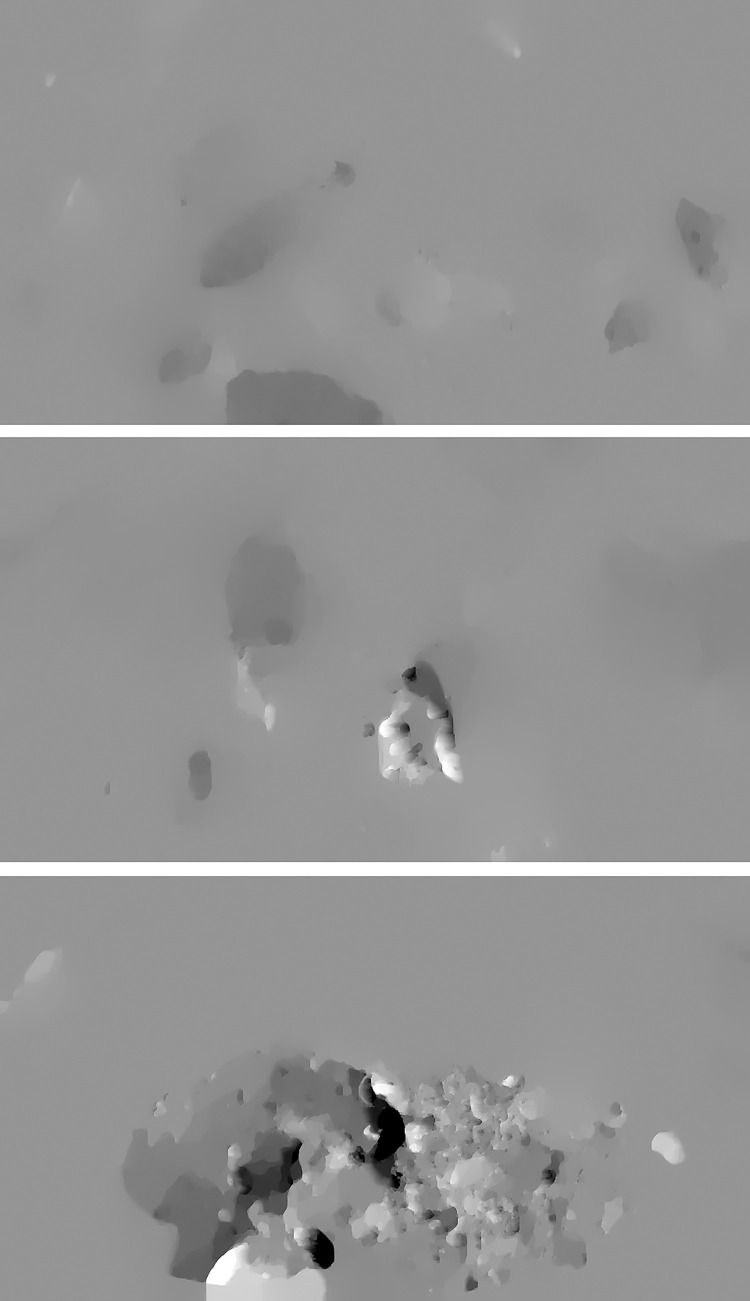
Optical flow images of three different feeding states. (A) None Feeding State. (B) Weak Feeding State. (C) Strong Feeding State.

Optical flow [25] defines the instantaneous velocity of pixel motion for objects in spatial motion on the observational imaging plane. This method leverages pixel changes within an image sequence in the time domain. It scrutinizes the correlation between successive frames to establish correspondences between the preceding and current frames. This approach facilitates the computation of motion information for objects between successive frames. Typically, the instantaneous rate of change of grayscale intensity at a specific coordinate point on a two-dimensional image plane is characterized as an optical flow vector. All RGB images in the dataset underwent optical flow processing, amounting to 145,712 optical flow images. This set comprises 45,346 non-feeding states, 47,084 strong feeding states, and 53,828 weak feeding states. The optical flow images corresponding to each feeding state are depicted in Fig 4.

The fundamental constraint equation of optical flow operation is as follows:

Contemplating the light intensity of a pixel *I*(*x*, *y*, *t*) in the initial frame (where *t* denotes the time dimension) as it traverses a distance of (*dx*, *dy*) to reach the subsequent frame within a time interval of *dt*, the constancy assumption posits that, being the same pixel, the light intensity remains constant before and after the movement. This is expressed as:

I(x,y,t)=I(x+dx,y+dy,t) (1)

Taylor expansion is performed on the right end of Eq (1) to obtain:

$$I(x,y,t)=I(x,y,t)+\frac{\partial I}{\partial x}dx+\frac{\partial I}{\partial y}dy+\frac{\partial I}{\partial t}dt+\varepsilon$$ (2)

Where ε represents the second-order infinitesimal term, which is negligible. By dividing *dt* by Eq (2) after Eq (1) we can get the following:

$$\frac{\partial I}{\partial x}\frac{dx}{dt}+\frac{\partial I}{\partial y}\frac{dy}{dt}+\frac{\partial I}{\partial t}\frac{dt}{dt}=0$$ (3)

Let *u v* be the optical flow velocity vectors along the X-axis and Y-axis, respectively:

$$u=\frac{dx}{dt},v=\frac{dy}{dt}$$ (4)

Let $I_{x}=\frac{\partial I}{\partial x},\;I_{y}=\frac{\partial I}{\partial y},\;I_{t}=\frac{\partial I}{\partial t}$ and they represent the partial derivatives of the gray values of the pixels in the image along the X, Y, and T directions, respectively.

In summary, Formula (3) can be written as follows:

$$I_{x}u+I_{y}v+I_{t}=0$$ (5)

*Ix*, *Iy*, *It* can be derived from the image data, while (*u*, *v*) represents the optical flow vector.

**Fig 5:**
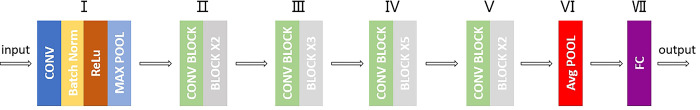
The basic structure of ResNet-50.

**Fig 6:**
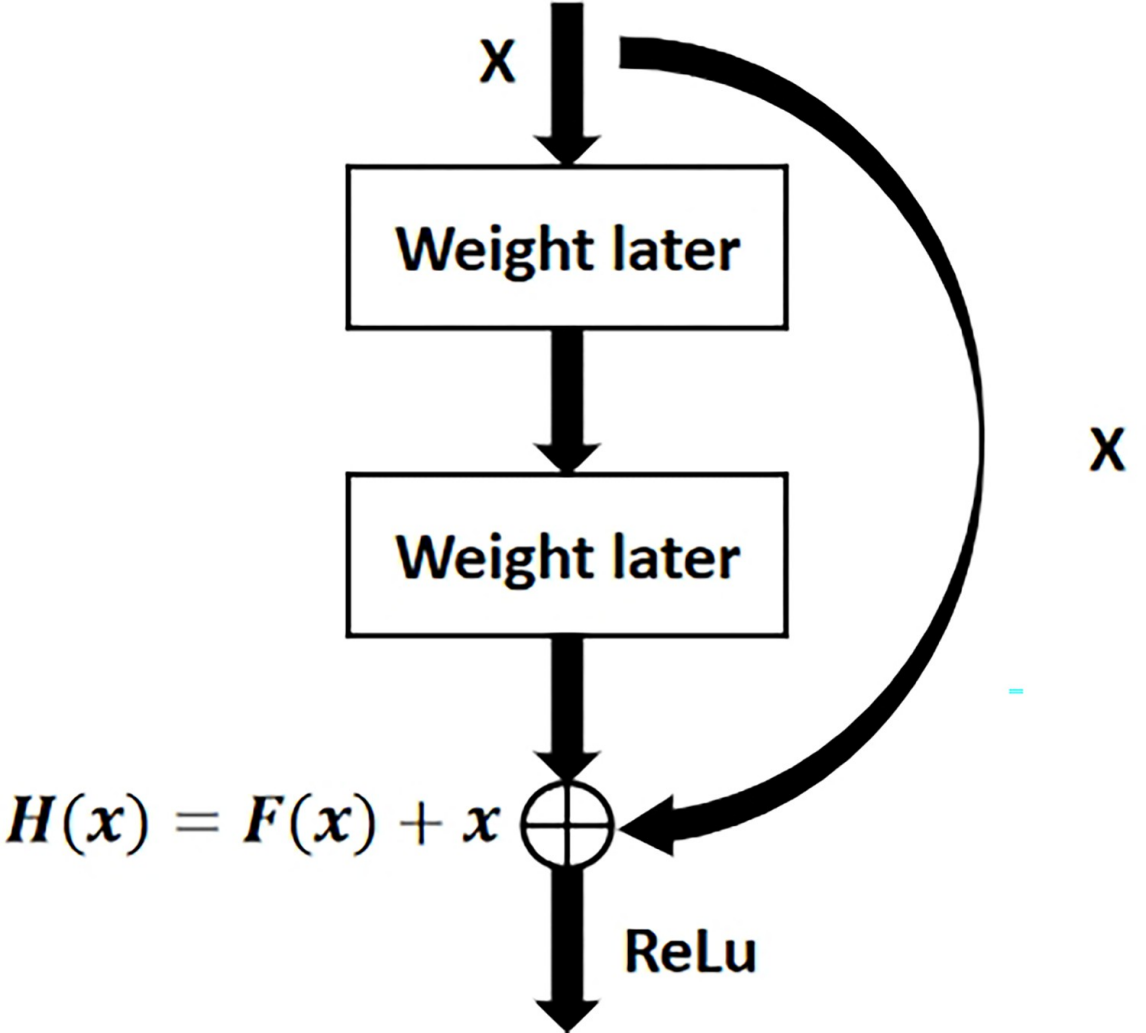
ResNet network residual unit structure.

The ResNet-50 [26] network is composed of 49 convolutional layers and a fully connected layer. As depicted in Fig 5, the architecture of the ResNet-50 network can be divided into seven sections. The initial section, devoid of residual blocks, primarily performs convolution, activation function, regularization, and maximum pooling operations on the input. The second to fifth sections of the structure encompass the configuration of the residual block. The green block in Fig 6 maintains the size of the residual block but alters its dimension. In the ResNet-50 network architecture, each residual block comprises three convolution layers, totaling 1+3×(3+4+6+3) = 49 convolution layers. Incorporating the final fully connected layer brings the total to 50 layers, hence the nomenclature of ResNet-50. The network processes an input of size 224×224×3, and following convolution computations in the initial five sections, the output transforms to 7×7×2048. The pooling layer converts it into a feature vector. Ultimately, the classifier processes the feature vector and generates the category probability as the output. The cornerstone of the ResNet lies in the structure of its residual unit, as illustrated in Fig 6. The residual network unit integrates a cross-layer connection. The curve in the graph facilitates the direct passage of the input across the layer, executing the exact mapping and adding it to the outcome of the convolution operation.

#### Classification network of spatial features based on binaryzation method

Based on the breeding environment of the experimental subjects and observations made during the feeding process, we discerned that fish induce varying degrees of water blooms during feeding. The size and extent of these water blooms can, to a certain extent, reflect the feeding intensity of the fish and serve as a spatial feature to assist in distinguishing the feeding intensity of the fish. Consequently, we utilize the binarization method to process the image, which serves as the input for the spatial feature classification network. Simultaneously, ResNet-50 is selected as the classification backbone network for training and accuracy testing. This strategy enables us to distinguish and categorize the feeding intensity of fish from a spatial perspective.

Image binarization [27] involves adjusting the gray value of all pixels to either 0 or 255, thereby transforming the entire image into a black-and-white representation. Binarization is performed using the average value of image pixels as the threshold for binarization processing. In a binary image, each pixel can only assume one of two values: pure black or pure white. Owing to the simplicity of binary image data, numerous visual algorithms depend on such representations. The use of binary images enhances the analysis of object shapes and contours. Various methods can be employed for binarization, with the threshold method (thresholding) being the most commonly utilized. This approach entails assigning pixel gray values greater than a specific critical threshold to the maximum gray value and those below this threshold to the minimum gray value, thereby achieving binarization. The formula is as follows:

$$f(x,y)=\left\{ \begin{array}{l} 1,\;(I_{S}(x,y)<T_{S}{\&I}_{V}(x,y)>T_{V}\\ 0,\;Other \end{array}\right.$$ (6)

**Fig 7:**
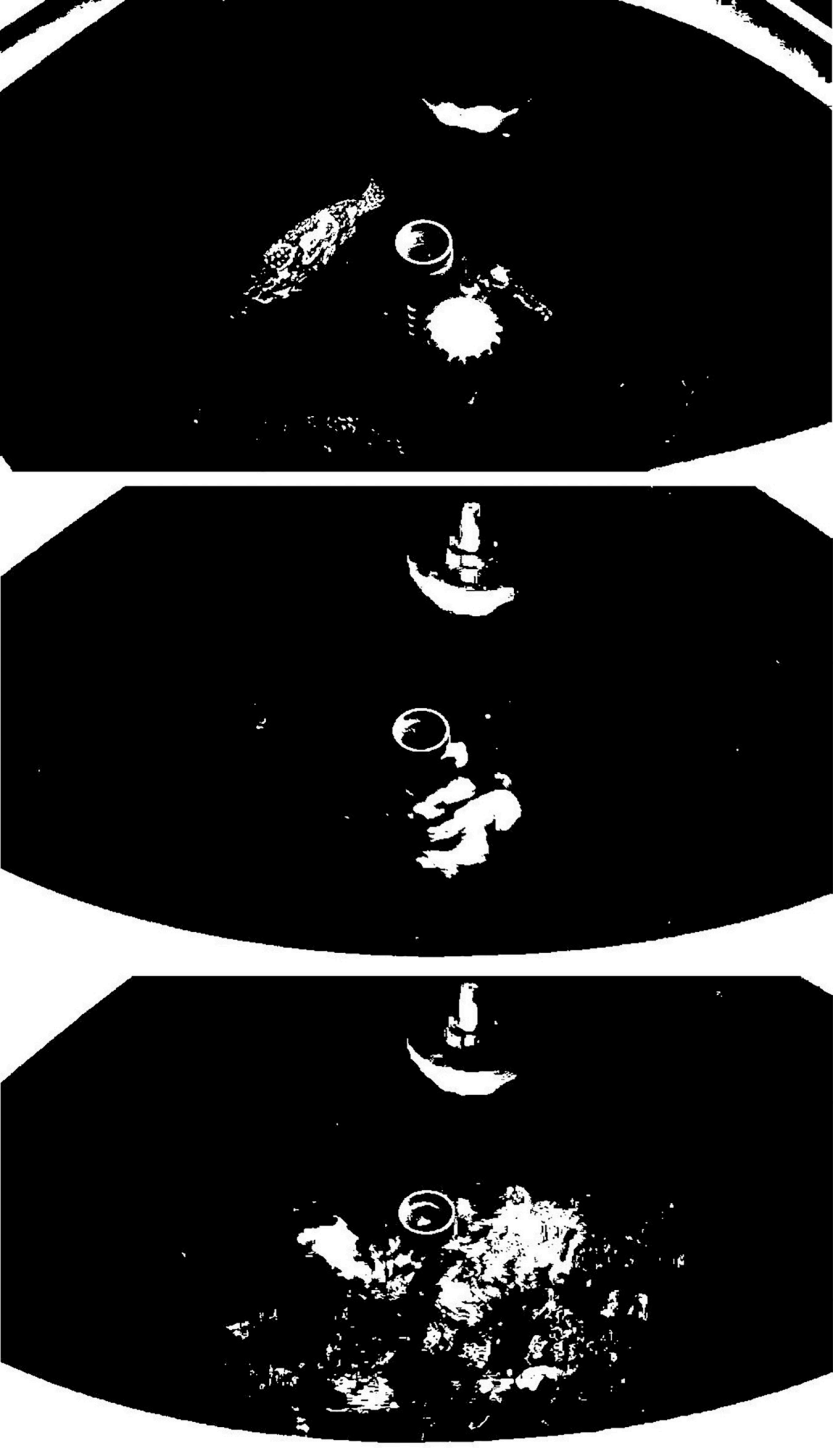
Binary images of three different feeding states. (A) None Feeding State. (B) Weak Feeding State. (C) Strong Feeding State.

All RGB images in the dataset underwent binarization, yielding 73,474 binary images. This set encompasses 22,888 non-feeding states, 23,728 strong feeding states, and 26,858 weak feeding states. The binary images corresponding to each feeding state are illustrated in Fig 7. From the diagram, it is apparent that the white areas mirror the various conditions of the reflective area on the water surface. This phenomenon transpires because the feeding process induces splashing, leading to a variety of shapes and sizes of the reflective area. Consequently, leveraging this characteristic facilitates the discrimination and classification of the fish’s feeding state.

#### Classification network of data statistical feature based on gray-level co-occurrence matrix (GLCM)

**Fig 8:**
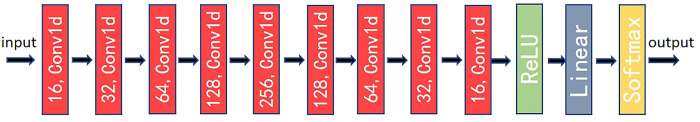
Basic structure of the one-dimensional convolutional neural network.

Our methodology entails determining the feeding intensity predicated on fish feeding videos, as the texture feature data of the image can efficaciously reflect the various alterations in the video. We employ the gray-level co-occurrence matrix to extract the energy, entropy, contrast, and correlation of the image, which serves as the input for the image texture feature classification network predicated on data statistics. Given that the data extracted by the gray level co-occurrence matrix is a one-dimensional array, we opt for a one-dimensional convolutional neural network as the backbone network. One-dimensional convolution solely convolves along the width and does not involve height convolution. We utilize a custom convolution combination to construct a classification network predicated on data statistics of image texture features to distinguish the feeding intensity of fish. The network structure is depicted in Fig 8.

The statistical method known as the Gray-Level Co-occurrence Matrix (GLCM) [28], introduced by R. Haralick and colleagues in the early 1970s, epitomizes a generalized texture analysis technique. It operates on the principle that the spatial distribution relationships among pixels in an image inherently encapsulate valuable texture information. The GLCM serves as a pivotal technique for analyzing the inherent texture features of images, celebrated for its robustness and stability. The gray-level co-occurrence matrix is established based on the pixel (*x*, *y*) with the image gray value of *i*. It computes the frequency *P*(*i*, *j*, *d*, *θ*) of the pixel (*x*+*a*, *y*+*b*) at a distance d with the gray value of *j*. Its mathematical expression is:

$$P(i,j,d,\theta )=\{\left[\begin{array}{c} (x,y),\frac{x+a,y+b}{f(x,y)}=i,\\ f(x+a,y+b)=j \end{array}\right]\}$$ (7)

Among them, *θ* represents the orientation of the gray-level co-occurrence matrix, typically taking four directions: 0°, 45°, 90°, and 135°.

In this study, the image’s gray level was quantized into 8 distinct levels, with a pixel distance set at 10. The pixel direction, denoted as θ, was considered at 0°, 45°, 90°, and 135°. A set of 16 eigenvalues, derived from four statistical measures—energy, entropy, contrast, and correlation, were employed as characteristic metrics to quantify the intensity of fish-feeding activity. The definitions and computational formulas for each feature are as follows:

1. Energy

Energy indicates the uniformity in the distribution of image gray levels and the overall texture thickness. A low energy value suggests intricate image texture when the element values in the gray-level co-occurrence matrix are akin. The resulting energy value is high if specific values within the gray-level co-occurrence matrix are large while others are comparatively small. A high energy value signifies a more uniform and regular texture pattern.


$$Asm=\sum_{i}\sum_{j}{P(i,j)}^{2}$$ (8)

2. Entropy

Entropy is a metric that quantifies the degree of randomness in the information content within an image. The entropy value reaches its maximum when all the values in the co-occurrence matrix are equal or when the pixel values demonstrate maximum randomness. Therefore, the entropy value serves as an indicator of the complexity of the gray distribution in the image. An increase in entropy value corresponds to heightened image complexity.


$$Ent=-\sum_{i}\sum_{j}P(i,j)\log\;P(i,j)$$ (9)

3. Contrast

Contrast is a metric that assesses the distribution of values in the image matrix and the extent of local variations within the image. It reflects both the clarity and the depth of the image texture. A higher contrast corresponds to deeper grooves in the image texture, yielding a more precise visual effect. Conversely, a slight contrast value implies shallow grooves in the image texture, leading to a blurred visual effect.


$$Con=\sum_{i}\sum_{j}{\left(i-j\right)}^{2}P(i,j)$$ (10)

4. Correlation

Indeed, correlation is employed to assess the similarity of gray levels within the image along the row or column direction, making it a crucial metric.


$$Corr=[\sum_{i}\sum_{j}(\left(ij)p(i,j\right))-\mu_{x}\mu_{x}]/\sigma_{x}\sigma_{y}$$ (11)

According to the above formula, different gray-level co-occurrence matrices will be obtained for different directions, gray levels, and pixel distances. Therefore, we can use the convolutional neural network to classify these values extracted from the image. By analyzing the statistical characteristics of the data of the graphic texture, accurate discrimination of the feeding state intensity of the cultured fish is realized.

### Experimental evaluation index

In this investigation, the performance of the fish feeding intensity discrimination method was evaluated using recall, accuracy, and the F1 score as metrics. Accuracy represents the capability to correctly identify various behaviors and is defined as the ratio of correctly identified samples to the total sample count. Precision reflects the proportion of accurate predictions among all instances predicted to belong to a specific class. Recall represents the proportion of items correctly classified out of those that necessitate classification. These metrics can be defined as follows:

$$Accuracy=\frac{TP+TN}{TP+FN+FP+TN}\times 100\%$$ (12)

$$Recall=\frac{TP}{TP+FN}\times 100\%$$ (13)

$$F1=\frac{2\times Precision\times Recall}{Precision+Recall}$$ (14)

Among them, true positive (TP), false positive (FP), false negative (FN), and true negative (TN) indicate that:

TP (true positive): The positive class is correctly identified as the positive class.

FP (false positive): The negative class is incorrectly identified as the positive class.

FN (false negative): The positive class is incorrectly identified as the negative class.

TN (true negative): The negative class is correctly identified as the negative class.

Note: Samples belonging to a certain class are typically referred to as positive samples, while those not belonging to that class are referred to as negative samples.

## Experimental results

### Results of three-stream network training and classification of fish feeding intensity

**Fig 9:**
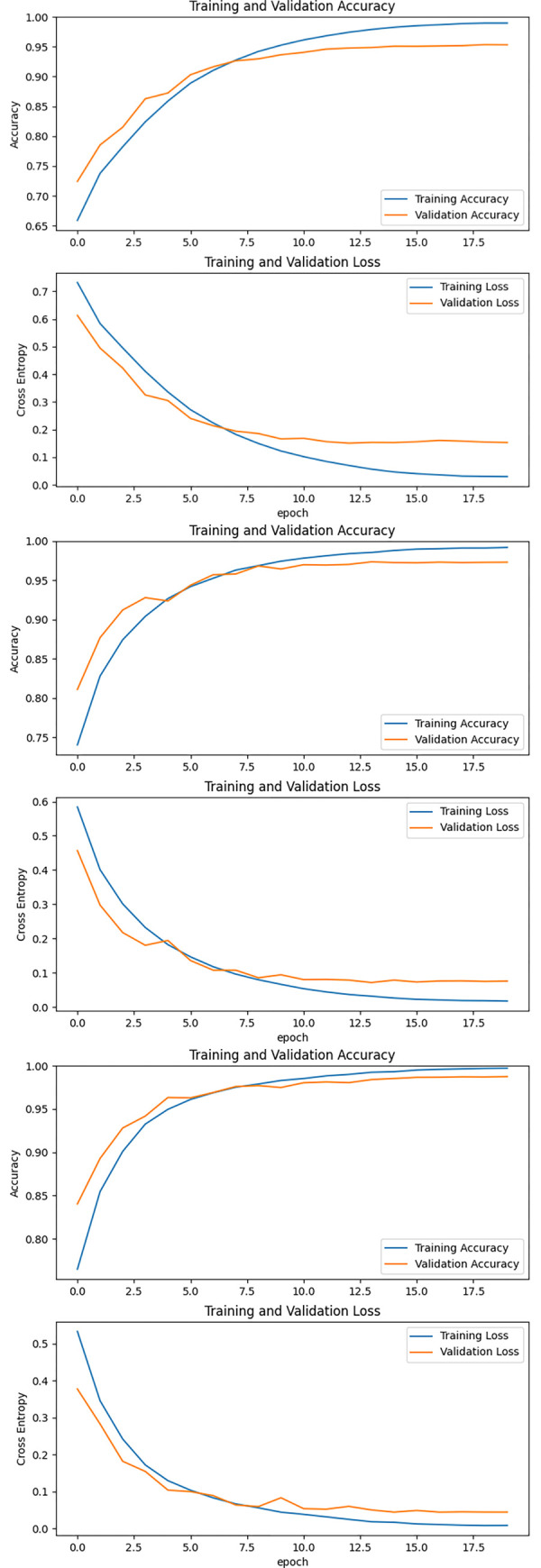
The training and testing results for the temporal feature network (A), spatial feature network (B), and data statistical feature network (C).

The same training strategy was employed to train and validate the three independent networks within the three-stream network, culminating in the results depicted in Table 2. Three evaluation indices—recall rate, F1 score, and accuracy—were utilized to validate the network’s training outcomes. Fig 9(A) presents the training and testing outcomes of the classification network predicated on temporal features. Post 20 training epochs, the loss for both the training and validation sets demonstrated a declining trend, ultimately stabilizing. The accuracy consistently augmented in the training set, albeit it manifested fluctuations in the validation set. Both displayed an ascending trend and eventually reached a steady state. The final outcomes were a loss of 0.112, an F1 score of 94.1%, a recall rate of 94.6%, and an accuracy rate of 95.8%. Fig 9(B) presents the training and testing results for the classification network based on spatial features. Similarly, after 20 training epochs, the loss for both the training and validation sets showed a decreasing trend and eventually stabilized. The accuracy consistently improved in the training set, while it exhibited fluctuations in the validation set. Both displayed an ascending trend and eventually reached a steady state. The final outcomes were a loss of 0.035, an F1 score of 98.1%, a recall rate of 98.1%, and an accuracy rate of 98.6%. Fig 9(C) illustrates the training and testing outcomes of the classification network predicated on data statistical features. Following 20 training epochs, the loss and accuracy of both the training and validation sets followed a similar pattern as the initial two epochs, decreasing initially and then stabilizing. The final results were a loss of 0.044, an F1 score of 97.5%, a recall rate of 97.7%, and an accuracy rate of 98.2%.

**Table 2:** Network training results.

Model	Loss	F1	Recall	Accuracy
Temporal Features Classification Network	0.112	94.1%	94.6%	95.8%
Spatial Feature Classification Network	0.035	98.1%	98.1%	98.6%
Data Statistical Features Classification Network	0.044	97.5%	97.7%	98.2%

**Fig 10:**
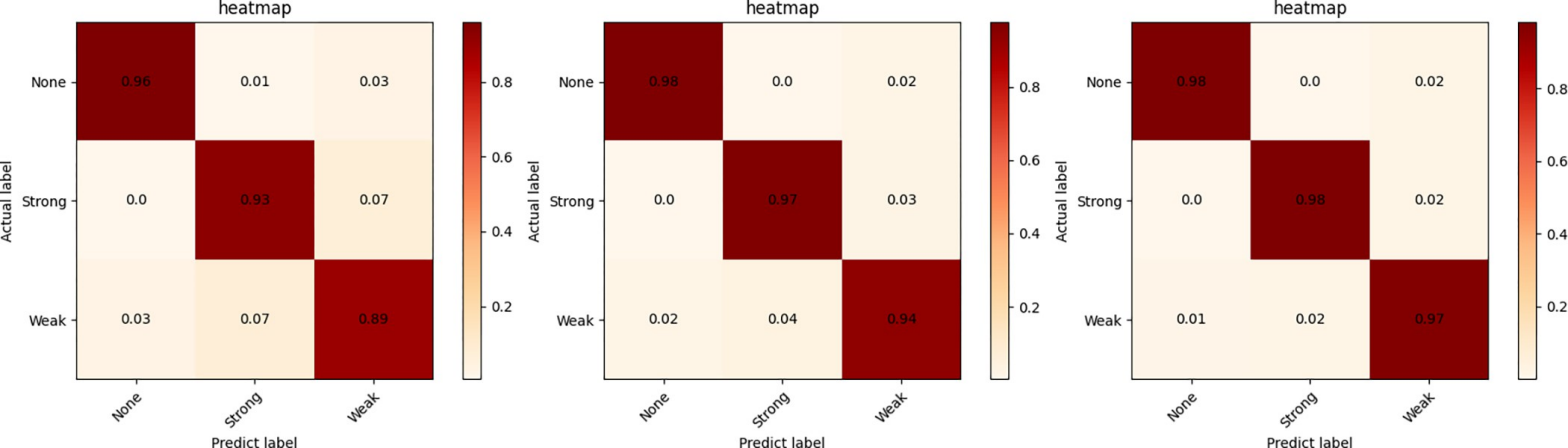
Three confusion matrix diagrams depicting the test results based on temporal feature network (A), spatial feature network (B), and data statistical feature network (C).

The test set images were evaluated using the optimally trained three-stream network to discriminate the feeding behavior of farmed fish. Table 3 showcases the performance evaluation metrics for each independent network and the overall network post fusion. In the test results of the classification network based on temporal features, the F1 score was 89.6%, the recall rate was 90.6%, and the accuracy rate was 92.5%. Fig 10(A) illustrates a confusion matrix derived from the classification outcomes of each image in the validation set. The recognition accuracy for the non-feeding state was 97%, for the strong feeding state was 95%, and for the weak feeding state was 93%. Additionally, 3% of the non-feeding state was erroneously classified as weak, and 4% of the strong feeding state was incorrectly classified as weak. Additionally, 2% of the weak feeding state was misclassified as a non-feeding state, and 5% was misidentified as a strong state. In the test results of the classification network based on spatial features, the F1 score was 94.4%, the recall rate was 95%, and the accuracy rate was 96%. Fig 10(B) presents a confusion matrix derived from the classification outcomes of each image in the validation set. The recognition accuracy for the non-feeding state was 98%, for the strong feeding state was 97%, and for the weak feeding state was 94%. Furthermore, 2% of the non-feeding state was misclassified as weak, and 3% of the strong feeding state was incorrectly classified as weak. Furthermore, 2% of the weak feeding state was misidentified as a non-feeding state, and 4% was misclassified as a strong state. In the test results of the classification network based on data statistical features, the F1 score was 95.3%, the recall rate was 95.6%, and the accuracy rate was 96.3%. Fig 10(C) presents a confusion matrix derived from the classification outcomes of each image in the validation set. The recognition accuracy for the non-feeding state was 98%, for the strong feeding state was 98%, and for the weak feeding state was 97%. Moreover, 2% of the non-feeding state was mistakenly identified as weak, and 2% of the strong feeding state was misclassified as weak. Additionally, 1% of the weak feeding state was misidentified as a non-feeding state, and 2% of the weak feeding state was misclassified as a strong feeding state.

**Table 3:** Evaluation index of classification results of fish feeding status by three-stream network.

Model	F1	Recall	Accuracy
Temporal Features Classification Network	89.6%	90.6%	92.5%
Spatial Feature Classification Network	94.4%	95.0%	96.0%
Data Statistical Features Classification Network	95.3%	95.6%	96.3%
**The integrated Three-Stream network**	**98.7%**	**98.9%**	**99.3%**

The performance of the network, post the integration of the three independent networks, is summarized in Table 3. The fusion method employed was the voting method, which resulted in an F1 score of 98.7%, a recall rate of 98.9%, and an accuracy rate of 99.3%. To further assess the integrated three-stream network, a new dataset, not utilized during the training process, was selected for testing. We randomly selected 300 images from each feeding state (strong, weak, and none), totaling 900 images for network performance testing. The results indicated that out of the non-feeding state images, 299 were accurately identified, with one non-feeding state image misclassified as a weak feeding state image. Additionally, out of the strong feeding state images, 298 were accurately identified, and two were misclassified as weak feeding state images. All 297 images of the weak feeding state were accurately identified. One image of the weak feeding state was misclassified as a non-feeding state image, and two images of the weak feeding state were identified as strong feeding state images. The F1 score, recall rate, and accuracy of the model all exceeded 98%, affirming the effectiveness of our proposed method.

### Ablation study

To systematically assess the independent and combined effects of fish feeding intensity discrimination networks predicated on different features within the three-stream network model of fish feeding intensity discrimination, we conducted ablation studies. The same training strategy was employed to train and test various network combinations. The impact of each combination on the model’s performance was thoroughly analyzed using metrics such as the F1 score, recall, and accuracy.

For different network combinations, as depicted in Table 4, we investigated seven distinct network structure combinations. The table outlines the performance of each combination network in the task of discriminating fish feeding intensity. When the training strategy remains consistent, the optimal outcome is achieved by the three-stream network model of feeding intensity discrimination, which is based on temporal, spatial, and statistical characteristics. The F1 score, recall, and accuracy are 98.7%, 98.9%, and 99.3%, respectively. This result suggests that the best outcomes are obtained when considering the three characteristics of fish feeding intensity. Moreover, this indicates that the model can provide technical support for discriminating fish feeding intensity.

**Table 4:** Ablation study.

Image Processing Method			Evaluating Indicator		
MethodⅠ	MethodⅡ	MethodⅢ	F1	Recall	Accuracy
Optical Flow	-	-	89.6%	90.6%	92.5%
-	Binarization	-	94.4%	95.0%	96.0%
-	-	GLCM	95.3%	95.6%	96.3%
Optical Flow	Binarization	-	95.0%	95.2%	96.5%
Optical Flow	-	GLCM	95.2%	95.7%	96.6%
-	Binarization	GLCM	96.6%	97.5%	98.1%
Optical Flow	Binarization	GLCM	**98.7%**	**98.9%**	**99.3%**

### Comparative experiment

To validate the practical applicability of our proposed method for discerning feeding intensity, we conducted a comparative analysis using our dataset against several other established methods. In Table 5, these methods include the LightGBM model utilizing Lucas-Kanade (LK) optical flow technology [29], the Support Vector Machine (SVM) model based on the Gray-Level Co-occurrence Matrix (GLCM) [30], ResNet-101 [31] which has shown promising results in target recognition of labeled fish images, EfficientNet [32] which is used as the backbone network of the fish intelligent recognition system, and the 3D Convolutional Neural Network (C3D) model [33] specifically designed for three-dimensional convolution of video data. Our proposed model outperforms traditional computer vision methods that manually extract features using LightGBM and SVM models, with improvements of 6.0% and 26.1% respectively. Moreover, the CNN model based on image classification significantly surpasses traditional computer vision methods. Our method also exhibits certain advantages over the CNN models ResNet-101 and EfficientNet used for image classification, with improvements of 1.7% and 2.1% respectively. When compared to the C3D model, which also uses video as input, our method shows a 4.1% improvement. Furthermore, in terms of F1 score and recall rate, our proposed method outperforms other methods in discerning feeding intensity.

**Table 5:** Comparison with other models for fish feeding behavior discrimination.

Model	F1	Recall	Accuracy
LightGBM	92.5%	91.9%	93.3%
SVM	71.1%	71.8%	73.2%
ResNet101	96.4%	96.7%	97.5%
EfficientNet	96.1%	96.5%	97.2%
3D Convolution (C3D)	93.6%	93.4%	95.2%
**Our Method**	**98.7%**	**98.9%**	**99.3%**

Through comparative experiments, we find that our proposed method, which integrates computer vision with CNN, not only enhances accuracy but also eliminates the complex operation of manual feature extraction, compared to traditional computer vision methods. Compared to the CNN method based on image classification, our method, which processes videos using optical flow, takes into account the temporal features in the video sequence and improves accuracy. Compared to the C3D model, which also uses video as input, our method achieves higher accuracy by extracting more features from the video. In summary, our proposed model employs computer vision techniques to process video images, considering the temporal, spatial, and data statistical features of the video, and uses CNN as the main classification network. This model exhibits excellent performance in distinguishing the feeding intensity of farmed fish. These results profoundly demonstrate the accuracy and practicality of our proposed method in discerning the feeding behavior of farmed fish.

## Discussions

### Model credibility verification

**Fig 11:**
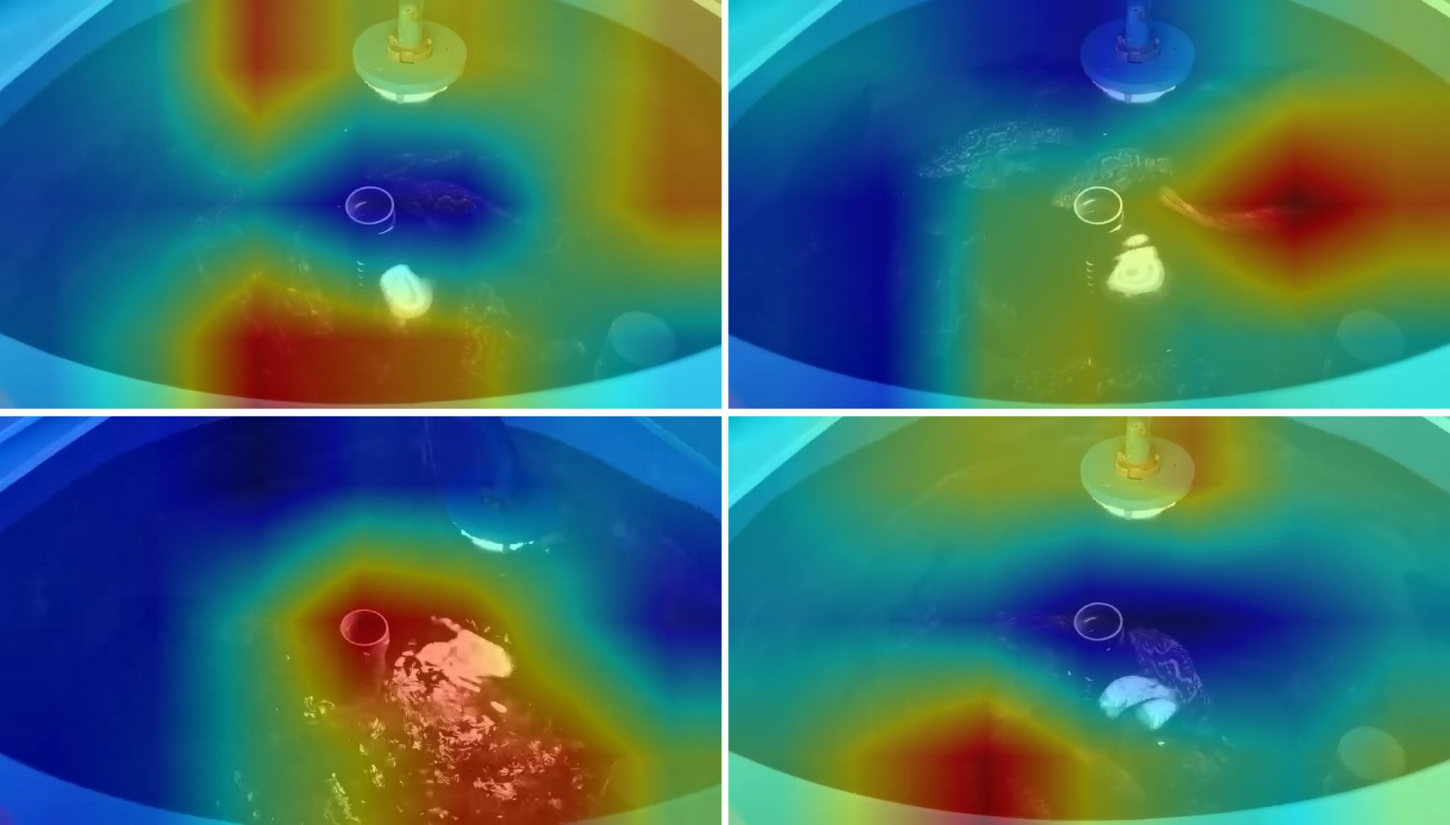
Class activation mapping (CAM) of test samples. (A), (B), and (C) are samples with correct predictions, classed as None, Weak, and Strong. (D) denotes the misclassified sample. Its essential actual value is Weak, and the predicted values are None.

In this study, the Class Activation Map (CAM) [34] method was employed to verify the credibility of the model. The CAM method confirms that the model’s classification is based on specific features, and it represents the features the model focuses on using different colors, ranging from light to dark. The deeper red indicates that the model pays more attention to the relevant features in that region, while blue indicates that the model does not focus on that area. Misclassified samples were collected and a visual analysis was performed using the CAM method. The results are depicted in Fig 11. Three samples with correct predictions and one with an incorrect prediction were randomly selected from the test dataset. In Fig 11(A), the model’s attention is primarily focused on the water surface of the entire breeding pool. This observation indicates that the water surface is calm, the fish are not active, there is no feeding state, and the current feeding state is None. In Fig 11(B), the model’s attention is mainly focused on the water surface of the half-rearing pond. This observation suggests that some fish are gathered due to eating, and the current feeding state is Weak. In Fig 11(C), the model’s attention is primarily concentrated in the same small area. This observation indicates that the current fish swarm is highly concentrated in the feeding area due to the incentive to prey, and the current feeding state is Strong. In Fig 11(D), the actual feeding state of the fish in the image is Weak, but the model classifies it as None. Through Fig 11(D), we can observe that the model’s attention is focused on the barrel wall of the breeding barrel and the LED lamp used to supplement the light.

In conclusion, the Class Activation Map (CAM) method is employed to visualize the areas where the model focuses its attention, thereby validating that the model genuinely concentrates on the three crucial features of None, Weak, and Strong. This validation bolsters the credibility of the model’s classification. Furthermore, background subtraction technology can be utilized to rectify the issue observed in Fig 11(D), where the model’s attention is focused on other parts of the background, leading to discrimination errors.

### Performance of discriminant model for feeding behavior of cultured fish

The proposed model for discriminating feeding intensity in farmed fish was trained, validated, and tested on an 11th Gen Intel® Core™ i7-11800H @ 2.30GHz CPU and NVIDIA GeForce RTX 3060 Laptop GPU. During the model training phase, the model consumed approximately 8 GB of memory, utilizing 50% of the total 16 GB memory capacity available on the computer. The training process covered 20 epochs, with a total training time of 12.6 hours. The testing phase, which involved 1,798 images, took 93 seconds, as indicated in Table 6. This table provides insights into the work efficiency of different feature classification network models during training and testing. On average, the recognition time for each image was approximately 0.052 seconds, satisfying the requirements for real-time recognition. The model size was 90 MB, making it easily applicable in the field of aquaculture.

**Table 6:** Comparison of processing time of different feature network models.

Model	Training Efficiency	Testing Efficiency
Temporal Features Classification Network	17.03it/s	19.87it/s
Spatial Feature Classification Network	20.00it/s	19.30it/s
Data Statistical Features Classification Network	19.23it/s	20.15it/s

As a result, the model showed commendable performance in terms of memory utilization, training duration, testing duration, and overall model size. The proposed discriminative model for analyzing feeding behavior in cultured fish shows potential for deployment on edge devices, addressing the real-time recognition requirements for fish feeding behavior in aquaculture. Although there were challenges related to memory usage and training time on laptop devices, these issues can be mitigated by deploying the model on servers or devices with enhanced performance in practical aquaculture settings.

### Model applications

**Fig 12:**
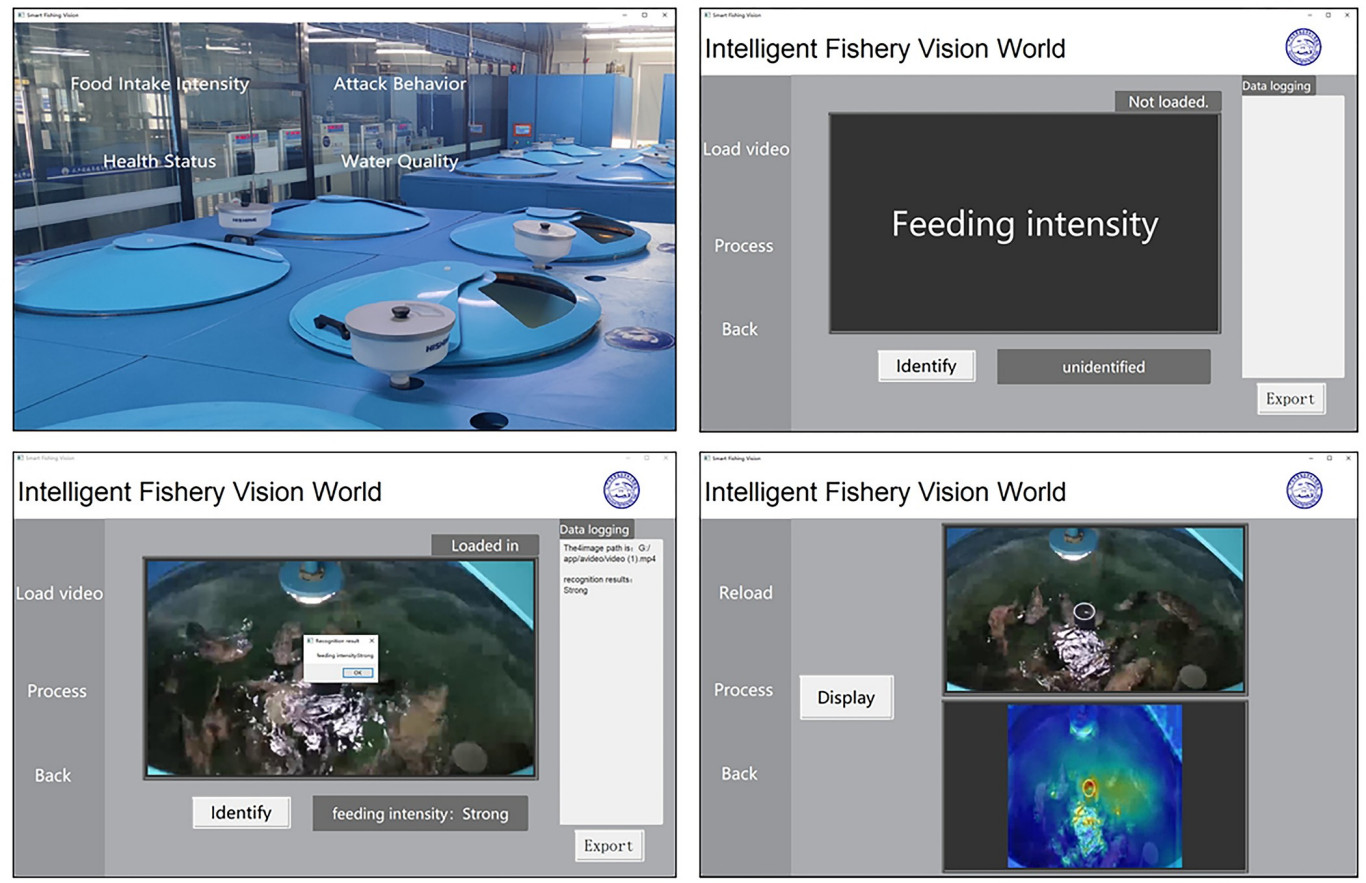
Software function interface.

To facilitate the practical implementation of the model, it has been utilized as the core technology to develop robust and practical software. The software interface, depicted in Fig 12, features a range of efficient functions that enable the easy importation of fish-feeding videos for identification. Through the recognition function, users can invoke the three-stream network model based on time series, spatial features, and data statistical characteristics, as proposed, to accurately assess the fish feeding intensity in the imported video. The software can generate the corresponding CAM (Category Activation Mapping) video. Additionally, users can employ the process analysis function to compare the original video with the CAM video. This function allows for a comprehensive understanding of the software’s focus throughout the entire recognition process, offering a reliable basis for staff to assist in analyzing and discriminating results. The application of this software goes beyond efficient and accurate fish-feeding status discrimination. It also contributes to intelligent and precise feeding practices in the aquaculture industry, leading to significant cost reductions, substantial improvements in economic benefits, and effective mitigation of water pollution issues. The software is a crucial technical support tool for the healthy growth and sustainable development of fish.

## Conclusions

In this study, we propose an innovative method to discern the feeding intensity of farmed fish by integrating computer vision technology with Convolutional Neural Networks (CNN). Our proposed three-stream network combines temporal, spatial, and data statistical features to evaluate the feeding intensity of farmed fish. During the training and validation stages, these three networks operate independently. However, during testing and practical application, their classification results are intelligently integrated to produce the final discriminative outcomes. By using images processed through computer vision technology as inputs to the CNN, we enhance the network’s focus on specific features during training. Simultaneously, we leverage CNN as the core classification network to significantly reduce time, computation, and labor costs. Ultimately, our proposed method achieves an impressive accuracy of 99.3%. Furthermore, we thoroughly validate the considerable superiority of our model by comparing it with alternative methods for discriminating fish-feeding states. We aim to implement this technology in practical production. Currently, this technology can discern feeding states on high-level GPU computers. To achieve industrialization, further algorithm optimization is necessary, and the integration of multiple functions is required to establish an intelligent monitoring system suitable for factory farming environments. This method should be applied to different growth stages of farmed fish for validation. This approach can lead to more precise and reliable discrimination results. Deploying the model to edge devices enables rapid and accurate discrimination under low computing power conditions. Once the technology matures, embedding it into plant protection drones capable of loading bait will facilitate intelligent and precise autonomous feeding in large water surface aquaculture environments. In this paper, the experiment is carried out in the recirculating aquaculture system. One of the advantages of the recirculating aquaculture system is that it can maintain water quality. Therefore, the water quality in this experiment is clearer and the image is clearer. In the actual breeding environment, it can be considered to increase the underwater camera or use the infrared camera for data acquisition to avoid the influence of water quality and other factors on the discriminant results.

In conclusion, the methodology presented in this study promotes a scientific and intelligent differentiation based on the feeding intensity of aquaculture fish in real-world production. This method can provide scientific support for optimizing feed cost savings. The research provides robust technical reinforcement for the dynamic expansion of aquaculture, establishing a crucial technical foundation for the healthy growth of farmed fish and the sustainable development of aquaculture.
